# Supplementary material for: Principal investigators over-optimistically forecast scientific and operational outcomes for clinical trials
Source: PLoS One. 2022 Feb 8;17(2):e0262862. doi: 10.1371/journal.pone.0262862 (PMC8824379; doi:10.1371/journal.pone.0262862)
Supplement: S1 File — 1) Repository for datafiles and codebook, 2) Survey, 3) Fig A1: Histograms of Brier scores, and 4) Sensitivity analysis including Fig A2: Histogram of Forecasts and Brier scores. Fig A3: Calibration curves from sensitivity analysis. Fig A4: discrimination from sensitivity analysis. (PDF) [file pone.0262862.s001.pdf]

APPENDICES

Principal Investigators Over-optimistically Forecast Scientific and Operational Clinical Trial Outcomes

Daniel M. Benjamin, Spencer P. Hey, Amanda MacPherson, Yasmina Hachem, Kara S. Smith, Sean X. Zhang, Sandy Wong, Samantha Dolter, David R. Mandel, Jonathan Kimmelman

1. REPOSITORY FOR DATAFILES AND CODEBOOK:
https://osf.io/67qn4/?view_only=679d89eccf48475092fe7aa26f235e76

2. SURVEY
Dear [NAME],
We are conducting a publicly funded study aimed at understanding researcher judgment in clinical trials. Our sampling method identified your trial "STUDY TITLE" (NCT#), so we invite you to answer four questions (about 5 minutes) about this trial.

When answering the questions below, please select a number between 0% and 100%. 0% means you are absolutely certain the event will not occur, and 100% means you are absolutely certain it will occur. A value of 50% means you think the event is as likely as not to occur.

To respond, reply to this email, or use our web-interface at the following link:
http://www.translationalethics.com/forecasting/response/?id=4479&code=2d039f07c9

1. What is the likelihood that your study will complete primary endpoint data collection by the date specified in the registry, [DATE]?

2. What is the likelihood that your study will demonstrate a statistically significant effect on [PRIMARY OUTCOME]?

3. What is the likelihood that your study will be successful in achieving its recruitment target of [N] patients at the point the study is officially closed?

4. What is the likelihood that 15% or more patients in the experimental treatment arm will experience a serious adverse event (grade 3-5) that is probably or definitely intervention-related?

Best regards,
[RESEARCH ASSISTANT]
STREAM Research Group
On behalf of Dr. Jonathan Kimmelman
McGill University

About this study: Our study has been approved by the McGill IRB. Your answers and the fact of your participation would be maintained in strict confidence. If you would like to decline participation, you can click the "decline" link below or e-mail us. Positive responses to this e-mail will be interpreted as consent. You can also visit our website [http://www.translationalethics.com/projects/forecast-study/] to learn more.

If you do agree to participate, we would also like to re-contact you for a 5-minute questionnaire after the study closes.
If you would like to decline to participate and/or receive no more emails on this subject, please click here:

3. BRIER SCORES

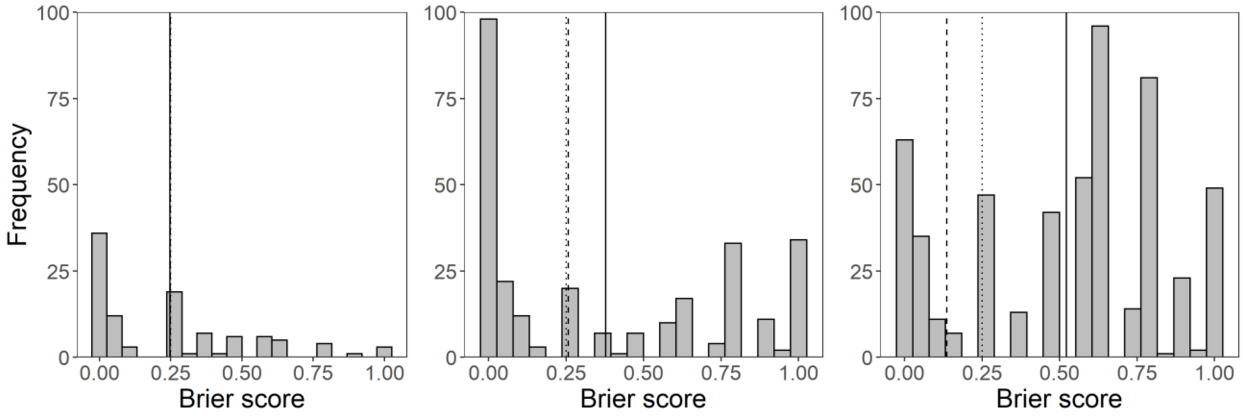

Figure A1: Histograms of Brier scores. Distribution of Brier scores for primary outcome attainment, completion date, and recruitment. The vertical solid line represents the mean, and the intermittent lines represent the prediction algorithms-uninformative (dotted) and base rate (dashed).

4. SENSITIVITY ANALYSIS

We performed a sensitivity analysis whereby only those trials for which we had all three outcomes were analyzed. The results of these analyses are presented below.

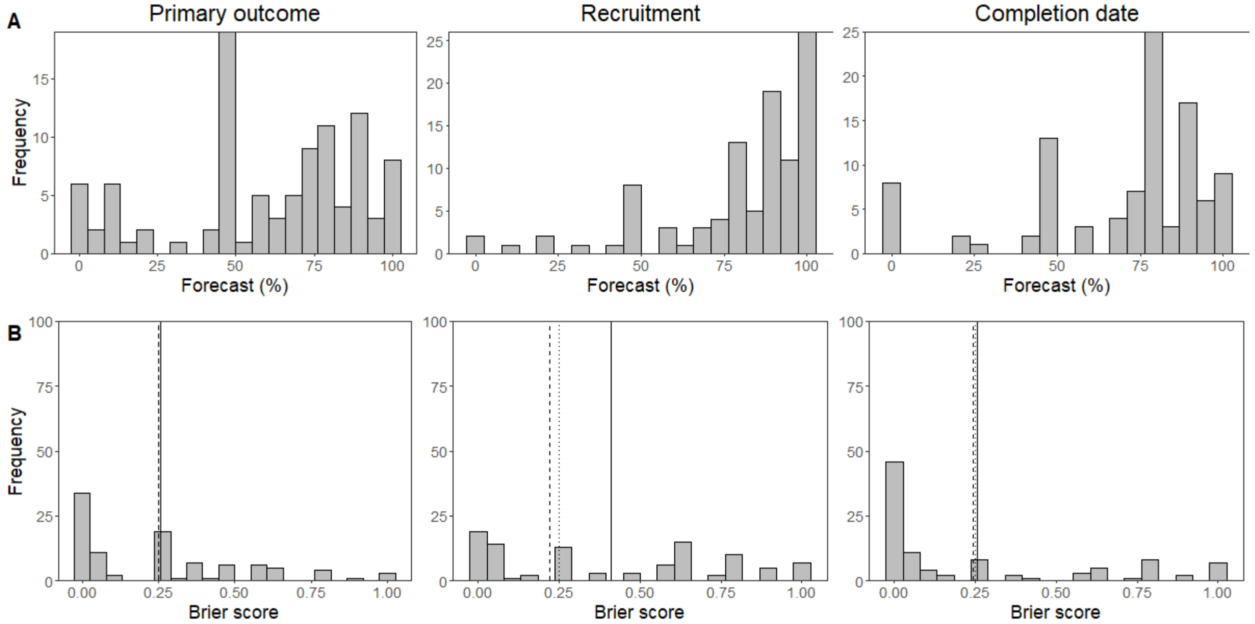

Figure A2: Histogram of Forecasts and Brier scores. A) Distribution of forecasts for primary outcome attainment, completion date, and recruitment. B) Distribution of Brier scores. The vertical solid line represents the mean, and the intermittent lines represent the prediction algorithms-uninformative (dotted) and base rate (dashed).

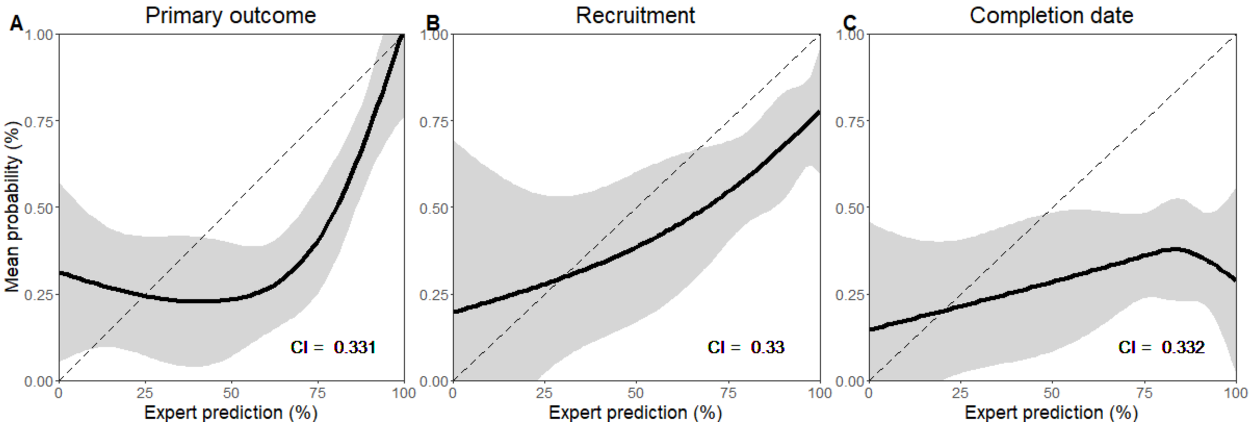

Figure A3: Calibration Curves from Sensitivity Analysis. Black lines show model-based (GLM) calibration with confidence region for N=104 trials with realized primary endpoints; CI = calibration index; dotted line is a slope = 1 benchmark.

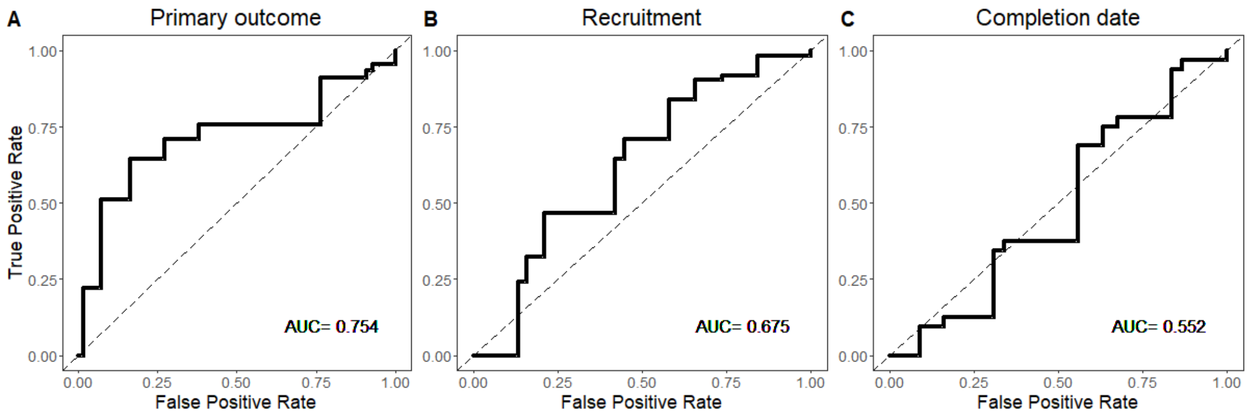

Figure A4: Discrimination from Sensitivity Analysis. Receiver Operator Characteristic Curves for N=104 trials with realized primary endpoints**;** AUC = area under the curve; dotted line is a slope = 1 benchmark.
