# Supplementary material for: Principal investigators over-optimistically forecast scientific and operational outcomes for clinical trials
Source: PLoS One. 2022 Feb 8;17(2):e0262862. doi: 10.1371/journal.pone.0262862 (PMC8824379; doi:10.1371/journal.pone.0262862)
Supplement: Attachment — Submitted filename: Review PlosOne RESP to REF 2021_CLEAN.doc [file pone.0262862.s002.doc]

EDITOR COMMENTS :

- In the abstract: please add a few words in the conclusion about the limitations in order to avoid any spin;

**>> We added the following to the conclusions: “They nevertheless showed modest ability to discriminate between positive and non-positive trial outcomes. Low survey response rates may limit the generalizability of our findings.”**

**>> Generalizability also addressed in the 4^th^ paragraph in the Discussion**

- I appreciate the transparency in the manuscript and in the appendix but I missed a mention of a pre-registration:

. If the study was registered, please provide a specific number of registration;

. If the study was not registered, please make it explicit;

**>> This study was not pre-registered as it was launched in 2013 before pre-registration was common in the social sciences (it is roughly consistent with our grant proposal). The Open Science Framework only launched in 2013 and Aspredicted at the end of 2015. This has been explicitly added to the Methods (p8).**

I would also suggest to edit the method section in order to move any change to the initial protocol in a dedicated paragraph "changes to the initial protocol".

**>> Since the study was not pre-registered, we did not undertake this.**

I agree with the reviewers that the paper is about an important issue.

**>> Many thanks**

Please submit your revised manuscript by Nov 29 2021 11:59PM. If you will need more time than this to complete your revisions, please reply to this message or contact the journal office at [plosone@plos.org](mailto:plosone@plos.org). When you're ready to submit your revision, log on to <https://www.editorialmanager.com/pone/> and select the 'Submissions Needing Revision' folder to locate your manuscript file.

**Reviewer's Responses to Questions**

**Comments to the Author**

1. Is the manuscript technically sound, and do the data support the conclusions?

Reviewer #1: Partly

Reviewer #2: Partly

Reviewer #3: Yes

2. Has the statistical analysis been performed appropriately and rigorously?

Reviewer #1: Yes

Reviewer #2: I Don't Know

Reviewer #3: Yes

3. Have the authors made all data underlying the findings in their manuscript fully available? The [PLOS Data policy](http://www.plosone.org/static/policies.action#sharing) requires authors to make all data underlying the findings described in their manuscript fully available without restriction, with rare exception (please refer to the Data Availability Statement in the manuscript PDF file). The data should be provided as part of the manuscript or its supporting information, or deposited to a public repository. For example, in addition to summary statistics, the data points behind means, medians and variance measures should be available. If there are restrictions on publicly sharing data—e.g. participant privacy or use of data from a third party—those must be specified.

Reviewer #1: No

Reviewer #2: No

**>> See below**

Reviewer #3: Yes

* Need to anonymize (de-identify) trial IDs and institutions

4. Is the manuscript presented in an intelligible fashion and written in standard English?

Reviewer #1: Yes

Reviewer #2: Yes

Reviewer #3: Yes

**Reviewer #1:** 
The paper describes a study based on an electronic survey to elicit subjective probabilities of PIs regarding three outcomes of their clinical trials: positivity of primary outcome, successful recruitment and timely trial completion.

I found this paper interesting, but I have identified a number of issues that have to be addressed before I would recommend to pursue publication.

General comments:

The PIs were asked to provide their subjective probabilities regarding the three outcomes of the study. It is well known that experts are often unable to provide precise numerical values for their probabilities (Ch 7 in O'Hagan et al, 2006). The paper lacks discussion of this fundamental issue and information on the probability precision ("number of bins") in the survey. Was it possible that a PI specified a probability of 0.69, say? The histograms in Figure 2 suggest that at least 20 bins (all multiples of 0.05) have been used. I am rather sceptical that PIs can state their subjective probabilities to this degree of precision.

**>> We appreciate that probability elicitation is incredibly intricate. We felt that, because clinical trials themselves are statistical beasts, eliciting probabilities from principal investigators would be reasonable. As well, we wanted to eliminate any barriers that would get in the way of study participation – for example- we attempted to use an elicitation platform that could be embedded in an email response and did not require clicking through to a website, since people receiving invites on mobile phone might not follow through.**

**>> As O’Hagan points out, there are settings where experts are well-calibrated and others where they are not (see the last paragraph of 4.2.4.1). We opted with a probability scale to avoid priming them to think about other trials as would be necessary if asking in a frequency format (ie extrapolating a larger reference class of trials) and to minimize the cognitive burden; medical experts should have familiarity with probabilities. A total of 97% (all except 30 out of 921) of expert forecasts we elicited were, in fact, divisible by 5, and the only exceptions were meaningful values like 1, 99, 33, and 67 implying experts were not overselling their precision. Importantly, our major findings suggest unrealistic optimism, rather than a lack of precision. Had we asked with a 10- or 20-point scale, its hard for us to see plausible grounds for why our results would be any different. For instance, given the ordinal nature subjective probability forecasts, we would still expect higher forecasts for events that are more likely to occur and vice versa. This illustrates the importance of decomposing Brier scores. Calibration would suffer if the scale was cognitively mismatched with expert beliefs, but the ability to discriminate between positive and non-positive results would not.**

**>> to clarify the question of how probabilities were elicited (e.g. binning), we added the following statement to our methods section: “Probabilities were elicited by asking for a value between and inclusive of 0% and 100%. »**

The response rate (16.7%) is very low, although the survey was "designed to take no more that five minutes". Obviously there is a severe risk of bias. In response to this issue, the authors state on p. 16 that "our respondent sample was similar to a random sample of non-respondents". However, the second sample is a "random sample" of size 100 from a total of 4443-740=3703 trials, so the numbers quoted in the last column of Table 1 should be given with confidence intervals. I guess there will be large uncertainty for some of the numbers. For example, assuming an exponential distribution for the h-index with median 25.5, the 95% CI for the population median based on a simulated sample of size 100 may range from 20 to 35 (See Altman et al "Statistics with Confidence", Chapter 5, for details on computation of CI for a median). I am not convinced that we can conclude that the population of non-responders is similar to the responders, that seems to require a larger sample size among non-responders.

**>> Thank you for the suggestions and citations. We agree that the generalizability of our findings may be limited by low response rates, and now state this more clearly (see edits to abstract, and two additions to our discussion). This is one of the enormous challenges associated with studying experts (who, by definition, are difficult to incentivize and have many demands on their time) and using clinical trials.**

**>> In the previous draft, we sampled non-respondents as a means of assessing response bias and presented a number of characteristics alongside our response data. While the two populations were not identical, we did not find obvious grounds for concern in terms of dissimilarities between the two samples.**

**>> At the suggestion of this referee, we added confidence intervals around the h-index estimates. The confidence interval around the median h-index of non-respondents (21.3, 29.8) contains the median h-index for respondents for each outcome measure. We also tested the correlation between h-index and Brier score to determine the degree to which one should be concerned with the make-up of our sample. We did not find that expertise (h-index) correlated with accuracy (Brier Scores), r_primary_=.03, p=.78, r_recruit_=.12, p=.04, r_completion_=.05, p=.22.**

**>> We also think our findings are important even if we had sampled the worst 15% of principal investigators (although our non-responder analysis suggests otherwise). Every principal investigator is responsible for the ethical, scientific, and operational integrity of their trial, and thus ought to possess nominal competence for planning their study. If it is the case that our study over-sampled less skilled investigators, it nevertheless remains the case that a large number of principal investigators confront major difficulty anticipating timelines, recruitment and outcomes for their studies.**

I also have an issue with the primary outcome of the study. The probability of a positive ("significant") finding is known as the power. The PI usually serves as an "advocate" who believes in the assumed treatment effect. If the study has been properly designed, then why should the PI not simply quote the assumed power of the study (usually 80 or 90%)? From that perspective, the numbers quoted in Table 2 (mean 60%, median 66%) are shrunken towards smaller values and perhaps already reflect some "clinical realism"?

**>> Thank you for providing us an opportunity to clear up a misunderstanding. Statistical power is not the probability that the primary endpoint is true. Power is the probability the trial will find evidence in favor of the primary endpoint (i.e. reject H_0_) if it were true. In other words, power is not the probability of a positive finding. It’s the chance of detecting a true positive (1 - false negative) outcome. If it were true that power reflected the probability the outcome were true, predicting clinical trial outcomes would be trivial. But clearly this is not the case.**

**>> We also think it is important to distinguish PI “advocacy” from PI unrealism. Any competent investigator working in a disease area like ALS, Alzheimer’s disease, or Parkinson’s disease will know that basically no drugs have yet been shown to have large disease modifying effects (i.e. base rates for positive trials for a disease modifying treatment in these areas are <5%). As such, a PI “advocate” who predicts a 30% chance of a positive result might seem pessimistic (i.e. 30% means that the trial is much more likely to be non-positive than positive), but is actually very optimistic relative to base rates.**

The poor predictions of "recruitment" and "completion date" are more worrying, it seems.

**>> We agree. We ask about both scientific and operational outcomes because they reflect two aspects of expert judgment in this setting. One goal of the design was to avoid conflating these two categories of outcomes. Trials can be non-positive either because the treatment under study does not work or because it is difficult to carry out a clinical trial. We have avoided judging which outcome is “more worrying.” We agree that planning is clearly biased, unrealistic, and should be considered for budgeting and funding agencies. One thing that is a bit reassuring is that flagging recruitment or timelines can at least be rectified, to some degree, with proper data monitoring.**

In general, I feel that Spiegelhalter (1986, Stat Med, 421-433, "Probabilistic prediction in patient management and clinical trials") might be a paper worth reading and citing. The calibration test for the Brier score could be added.

**>> Thank you for this suggestion (we love David Spiegelhalter’s work!). This citation has been added. We have run the additional calibration test as a sensitivity analysis which confirmed our previous results**.

There has been some recent success in predicting the outcome of replication studies (significance yes/no) using prediction markets (e.g. Dreber et al, PNAS, 2015). This involves not just one PI, but a whole group of experts to elicit probabilities for a positive trial result. I can see that this method is not directly applicable in the present context, but perhaps it would be worth to comment on.

**>> Thank you for this helpful suggestion. We are familiar with Dreber’s work and have added a sentence to the discussion referencing it.**

**>> However, it is important to point out that our study has very different aims than those of Dreber’s. Our study is about how P.I.s judge their own study. Unfortunately, there is not a critical mass of P.I.s for a given trial to run a prediction market of P.I.s. In this case, there should be no question about mismatched expertise or unavailable information. We do have other papers (see Benjamin et al, *Oncologist* 2020) that looks at pools of experts and in fact we find that only small numbers of experts are needed for substantial gains in accuracy**.

Minor comments:

Section 2.2: The process to select "a sample of newly registered clinical trials" seems rather complicated and error-prone. I am wondering whether some quality control has been implemented to make sure that all trials that met the inclusion criteria have been selected (e.g. double data entry through two independent investigators).

**>> The process of adding newly registered trials to our database was semi-automated, as detailed in the Trial Sample section of the Methods. We restricted inclusion to trials that were active and had a pre-recruitment status and used a standard invitation template. Clinicaltrials.gov is a bit of a jungle, containing many studies that do not use rigorous methodologies (e.g. open label studies, case series, “pay to participate” trials). We reasoned that assessing prediction skills for trials aimed at recruiting very small numbers, or with weaker methodologies, would be less interesting. We therefore included selection criteria aimed at quality control, like restricting inclusion to countries with effective trial oversight mechanisms and minimum sample size (≥ 20), etc. These criteria are enumerated in the first paragraph in the Trial Sample section; we added some text to explain inclusion criteria; we also neglected in the previous draft to note the minimum sample size (and we have now corrected this oversight).**

**>> As these criteria are objective (with some being automated), we did not implement double data entry through two independent investigators.**

p.5: The sentence starting with "Surveys were designed to take ..." is duplicated on p. 6.

**>> Thank you. We corrected this error.**

p.6: "random sample of 100 nonrespondents" How was the sample size determined? Related to this, what does "matched sample of 100 trials" in the caption to Table 1 mean? In which sense "matched"?

**>> This matched our targeted number of trials with realized primary endpoint outcomes. Because of the very long maturation periods between forecast and the possibility of verification, we decided, several years into the project, to perform analysis when 100 primary endpoint outcomes were realized. We added this clarification to the Methods.**

Table 1: I would add a row at the top describing the meaning of each column (e.g. "primary data available", etc).

**>> We agree. The headers got truncated during submission. This has been corrected.**

Table 2: "Extreme forecasts" I would also report the sum of the two rows "<=10%" and ">=90%" as this is what you quote in the text (37%, 62%, 40%).

**>> We added this row to Table 2.**

Table 2: "Brier scores": The expected Brier score depends on the underlying probability, even under the null hypothesis that the probabilities are the correct ones. From that perspective it doesn't make much sense to look at the Brier scores of the extreme forecasts, as we would expect the scores to be smaller than overall (which is in fact often, but not always the case).

**>> An expert forecasting 95% or 99% is stating her/his belief with near certainty. We wanted to probe prediction skill when PIs express extreme confidence. We expect the extreme BSs to be smaller if PIs are well-calibrated (or much greater if PIs are poorly calibrated. Our findings on this are inconclusive, though Table 2 suggests grounds for doubting experts who are highly confident about recruitment attainment and on-time trial closure**. **On the other hand- as the referee notes- when forecasters offer highly pessimistic projections, they seem to be well calibrated. We added a sentence in the discussion explaining this.**

Figure 2: More could be done to visualize the results. For example, the bars in row A could be splitted (with different colors) into outcomes that occur ("1") or not ("0"). I am also not sure whether we need to show the whole distribution of BS in row B, this seems quite unusual. It would also be good to use modern visualization tools, e.g. ggplot2 in R.

**>> Thanks for these suggestions. As for the second suggestion, we revised Fig2 accordingly. We now overlain forecasts as frequency polygons by outcome, and we eliminated our histogram of BS scores. Graphs were indeed made in ggplot2 in R.**

Figure 3: I found the bottom row in this figure not very informative. Smoothing in calibration plots usually depends a lot on the smoothing parameter and the distribution of the predictions. What about showing the more standard calibration curve with event frequencies against binned forecast probabilities with, say 10 bins?

**>> The model-based calibration curves we present are commonly used in forecast studies of this type (e.g. Benjamin et al, 2020; Mandel & Barnes, 2014). We made this choice to account for variability within the distribution because the sample sizes combined with the response patterns (the tendency for high confidence and extreme values) leads to some small, underrepresented bins. We have added a 5-bin non-smoothed calibration curve to each panel. (Note that the most aberrant points – bin 2 in primary outcome and recruitment - are the ones with the smallest bin sizes confirming our fears).**

I would suggest to add the AUC values (with CIs, please, also in the abstract!) and the calibration slope (again with CI) to Table 2. I am not sure if the "calibration slope" is the same as the "calibration index", please check. Seems like "calibration slope" is the standard,

e.g. Steyerberg's book "Clinical Prediction Models". Spiegelhalter's p-value from a calibration test based on the Brier score could be added, see the above paper for details.

**>> Thank you for these suggestions. We appreciate the importance of selecting appropriate measurements of accuracy. We have added AUC CIs to the abstract and Fig3.**

**>> The Murphy decomposition we use is pretty common in forecast studies (e.g. Bossavy, Girard, & Kariniotakis, 2013; Karoly et al, 2017; Wallsten, Budescu, & Zwick, 1991; Yates, McDaniel, & Brown, 1991). The calibration index is a more robust measure of calibration. Stevens and Poppe (2019) describe how the calibration slope can be misleading as a stand-alone measure. The calibration slope was originally suggested as a measure of spread, not calibration; the calibration slope can be difficult to interpret without additional information.**

**Also, our primary concern is performance relative to our naive benchmarks and relative to each other. Applying valid indicators to our outcomes and benchmarks reveals meaningful relative patterns, and changing calibration measures would not change the relative results.**

**>> Following the referee’s suggestion, we added Spiegelhalter’s calibration z-test to test if forecast magnitudes are about equal to proportion of observed outcomes. We obtained high z-values (p < .001) for all three outcomes confirming calibration was modest at best. This is now stated in the methods and results.**

**Reviewer #2:**

Benjamini and colleagues aimed to assess the accuracy of principal investigators’predictions about three events for their own clinical trials: positivity on trial primary outcomes, successful recruitment and timely trial completion. The topic is of interest but the manuscript needs major revisions before being considered for publication.

The main remark is the difficulty to read the results due to multiplicity and the unusual statistical methods used. The Brier score is well explained but the utility of the comparison of PI scores to predictions algorithms is difficult to understand. I wonder if a simpler analysis could show similar results. For example, for the primary endpoint, authors could show the percentage of studies for which PIs expected statistical significance and finally were negative.

**>> We thank the referee for these helpful suggestions. We appreciate that metrics used in our report, while commonplace in decision sciences and forecast literature, are not as well known in biomedicine, and have worked hard to explain metrics in simple language. We modified our description of algorithms to provide a bit more explanation and context. We hope this helps.**

**>> We like the suggestion of reporting the percentage of studies where PIs expected statistical significance, etc. and where the outcome was negative, and added this to our results section as the final sentence in the first paragraph of Section 3.3.**

Table 1: why adding columns 2 and 3 (with respective n= 281 and 536)? What is their utility regarding the aim of the study? In the same way why showing column 4 (match sample of 100 trials). This is confusing for me; the footnote does not mention the origin of the different columns.

**>> We regret our accidental omission of column headings in the submitted version and have corrected this. Each column represents the different trial outcomes for our study. The sample sizes varied for each outcome because primary endpoint (efficacy), recruitment, and completion by target end date, all mature at different rates. For example, we can record whether completion dates were hit by logging whether it has an active/recruitment status in clinicaltrials.gov on that date. In contrast, to find primary endpoint outcomes, we need to search for a public report in a publication or trial registry. The 4^th^ column is where we present a random sample of non-responders to test whether our sample is approximately representative of the population. We added a sentence at the end of our section 2.4 in the methods section. We hope this clarifies things.**

Fig 3 needs clarifications. At least, foot notes should be added. The results section says that when referring to figure 3, PI showed moderate discrimination for primary outcome but not for recruitment or timeline attainment. Please explain how you can say that when looking at the figure.

**>> Thanks for these suggestions. We Added AUC CIs both to Fig3 and the abstract. We also added some text to the Results section to help interpret discrimination findings**.

The utility of sensitivity analysis needs also to be clarified, especially the ROC curves shown in fig A3.

**>> As mentioned above, our three outcome measures mature at different rates. We ran the sensitivity analysis to test if the trials with realized primary endpoints are distinct from trials with forecasts that are still awaiting outcomes. One hypothesis is that trials that report results more quickly went better. They had fewer problems with recruitment, the outcome was positive, etc. Now explained more clearly in our methods section.**

Reviewer #3: The article is interesting and well-written. However, a think there is the room for a further improvement. In particular, it is well-known that there are several decompositions of the Brier score which provide a deeper insight. Therefore, I wonder if one of these decompositions may be useful to obtain further insights on the data analyzed by the authors.

**>> We are really heartened to hear the referee thinks our piece is interesting and well written.**

**>> Regarding Brier score, we decompose Brier by calibration and discrimination (see Figure 3). We use the Murphy decomposition because it is commonly used to assess subjective probability forecasts like our elicitation (e.g. Bossavy, Girard, & Kariniotakis, 2013; Karoly et al, 2017; Wallsten, Budescu, & Zwick, 1991; Yates, McDaniel, & Brown, 1991). We chose calibration and discrimination both because they represent unique and important components of forecast skill and because an audience of medical experts will be able to intuit these concepts. We’d certainly be happy to entertain other decompositions if the referee thinks one in particular could be of use in this context.**

Moreover, I have the additional minor remarks:
1. Captions of tables and figures are too long.

**>> We have shortened the captions considerably for Table 1 and Figure 2 moving some details to the Methods and Results body**.

2. In sentences like “timely closure for trials.(15)”, “metrics in prediction studies:(16) calibration”, and “measured using the calibration index;(13)(16) the”, the numbered citations should be placed before the punctuation.

**>> Fixed.**

6. PLOS authors have the option to publish the peer review history of their article ([what does this mean?](https://journals.plos.org/plosone/s/editorial-and-peer-review-process#loc-peer-review-history)). If published, this will include your full peer review and any attached files. If you choose “no”, your identity will remain anonymous but your review may still be made public.

**>> We consent to this**


**Do you want your identity to be public for this peer review?** For information about this choice, including consent withdrawal, please see our [Privacy Policy](https://www.plos.org/privacy-policy).

Reviewer #1: **Yes:** Leonhard Held

Reviewer #2: No

Reviewer #3: No

**>> Thanks to all three referees for their thoughtful reading of our manuscript and their many suggestions.**

[NOTE: If reviewer comments were submitted as an attachment file, they will be attached to this email and accessible via the submission site. Please log into your account, locate the manuscript record, and check for the action link "View Attachments". If this link does not appear, there are no attachment files.]

While revising your submission, please upload your figure files to the Preflight Analysis and Conversion Engine (PACE) digital diagnostic tool, <https://pacev2.apexcovantage.com/>. PACE helps ensure that figures meet PLOS requirements. To use PACE, you must first register as a user. Registration is free. Then, login and navigate to the UPLOAD tab, where you will find detailed instructions on how to use the tool. If you encounter any issues or have any questions when using PACE, please email PLOS at [figures@plos.org](mailto:figures@plos.org). Please note that Supporting Information files do not need this step.

--

Please include the following items when submitting your revised manuscript:

- A rebuttal letter that responds to each point raised by the academic editor and reviewer(s). You should upload this letter as a separate file labeled 'Response to Reviewers'.
- A marked-up copy of your manuscript that highlights changes made to the original version. You should upload this as a separate file labeled 'Revised Manuscript with Track Changes'.
- An unmarked version of your revised paper without tracked changes. You should upload this as a separate file labeled 'Manuscript'.

If you would like to make changes to your financial disclosure, please include your updated statement in your cover letter. Guidelines for resubmitting your figure files are available below the reviewer comments at the end of this letter.

If applicable, we recommend that you deposit your laboratory protocols in [protocols.io](http://protocols.io/) to enhance the reproducibility of your results. [Protocols.io](http://Protocols.io/) assigns your protocol its own identifier (DOI) so that it can be cited independently in the future. For instructions see: [https://journals.plos.org/plosone/s/submission-guidelines#loc-laboratory-protocols](https://journals.plos.org/plosone/s/submission-guidelines#loc-laboratory-protocols). Additionally, PLOS ONE offers an option for publishing peer-reviewed Lab Protocol articles, which describe protocols hosted on [protocols.io](http://protocols.io/). Read more information on sharing protocols at <https://plos.org/protocols?utm_medium=editorial-email&utm_source=authorletters&utm_campaign=protocols>.

We look forward to receiving your revised manuscript.

Kind regards,

Florian Naudet, M.D., M.P.H., Ph.D.
Academic Editor
PLOS ONE

Journal Requirements:

When submitting your revision, we need you to address these additional requirements.

1. Please ensure that your manuscript meets PLOS ONE's style requirements, including those for file naming. The PLOS ONE style templates can be found at

<https://journals.plos.org/plosone/s/file?id=wjVg/PLOSOne_formatting_sample_main_body.pdf> and <https://journals.plos.org/plosone/s/file?id=ba62/PLOSOne_formatting_sample_title_authors_affiliations.pdf>.

2. Please provide additional details regarding participant consent. In the ethics statement in the Methods and online submission information, please ensure that you have specified what type you obtained (for instance, written or verbal, and if verbal, how it was documented and witnessed). If your study included minors, state whether you obtained consent from parents or guardians. If the need for consent was waived by the ethics committee, please include this information.

3. We note that you have stated that you will provide repository information for your data at acceptance. Should your manuscript be accepted for publication, we will hold it until you provide the relevant accession numbers or DOIs necessary to access your data. If you wish to make changes to your Data Availability statement, please describe these changes in your cover letter and we will update your Data Availability statement to reflect the information you provide.

4. Please include captions for your Supporting Information files at the end of your manuscript, and update any in-text citations to match accordingly. Please see our Supporting Information guidelines for more information: <http://journals.plos.org/plosone/s/supporting-information.>
