# Supplementary material for: Principal investigators over-optimistically forecast scientific and operational outcomes for clinical trials
Source: PLoS One. 2022 Feb 8;17(2):e0262862. doi: 10.1371/journal.pone.0262862 (PMC8824379; doi:10.1371/journal.pone.0262862)
Supplement: Attachment — Submitted filename: Plos rev2 response.docx [file pone.0262862.s003.docx]

**PONE-D-21-10921R1**

**Dear Editor and Reviewer:**

**We appreciate the editor and referee offering suggestions to hone the analyses and messaging. See revisions below.**

Reviewer #1: Comments on the response to my comments on PONE-D-21-10921. The
numbers refer to the order of my original comments.

1) Thanks for giving more details on the elicitation procedure. Your
explanation indicates the presence of digit preference bias with all
percentages divisible by 5, except for 1, 99, 33 and 67. This should
be mentioned as a limitation of the study.

**>>We added a description of forecast granularity as a result in section 3.2 (Forecast Characteristics). We added further discussion of this finding in the limitations section of the Discussion. In this setting, we find this level of granularity is consistent with a lack of motivation of forecasters. We add references to Mellers et al (2015) and Tetlock & Gardner (2016) supporting the association between granularity and accuracy.**

3) Thank you for your explanation, I was inaccurate in my point. Yes,
the probability of a significant (positive) finding is

Pr(sig) = Pr(sig | H0) Pr (H0) + Pr(sig | H1) Pr(H1)

Now Pr(sig | H0) = usually 5% and Pr(sig | H1) is the assumed power
(say 80%). Therefore, if the clinician reports Pr(sig) it is possible
to infer her personal prior probability Pr(H0) (or equivalently Pr(H1)
= 1 - Pr(H0)). For example, if Pr(sig)=70% is reported by the
clinician we obtain Pr(H0) = 13% and so Pr(H1)=87%. Not sure if this
is helpful for the manuscript but I just wanted to make my point clear.

**>>Interesting. Thank you for your clarification. I take it this assumes our experts are coherent forecasters, of course (often not the case!)**

Minor point "Brier scores": Maybe I wasn't clear but I still think it
does not make sense to report Brier scores of extreme forecasts
because, under the null hypothesis of perfect calibration, the
expectation of the mean BS is (sum p_i*(1-p_i))/n, see Spiegelhalter
(1984, p. 427). If you only look at extreme forecasts (p_i very small
or very large), the mean BS will (under H0) therefore be on average
smaller than overall.

**>> We have removed these rows from Table 2.**

It seems that you need to enlarge the upper limit of ylim in Fig 2, as
one bar currently touches (or crosses?) the limit.

**>> We have fixed the margins of Fig2.**

General comment: The absence of a study protocol is a weak point. How
can we criticize the design and reporting standards of clinical trials
if our own standards are not any better? Surely, study protocols have
been very common in medical research prior to 2013. Not just for
clinical trials, but also for other study types (e.g. PRISMA was first
published in 2009).

**>>To be clear, we had a study protocol. This was/is required for IRB submission. We did not pre-register the hypotheses of this study, which is now fairly common practice in the social sciences, but was not at the time.**

Reviewer #2: I would like to thank the authors for their responses to reviewers comments and modifications that clarifed the manuscript and allow now its publication.

Reviewer #3: The authors did a good job in revising the paper according to the received comments. Therefore, the paper can be accepted as it is.

7. PLOS authors have the option to publish the peer review history of their article ([what does this mean?](https://nam10.safelinks.protection.outlook.com/?url=https%3A%2F%2Fjournals.plos.org%2Fplosone%2Fs%2Feditorial-and-peer-review-process%23loc-peer-review-history&data=04%7C01%7Cdbenjam1%40nova.edu%7C78fbbbb0f03d425fe55508d9c6151d09%7C2c2b2d312e3e4df1b571fb37c042ff1b%7C1%7C0%7C637758616263106991%7CUnknown%7CTWFpbGZsb3d8eyJWIjoiMC4wLjAwMDAiLCJQIjoiV2luMzIiLCJBTiI6Ik1haWwiLCJXVCI6Mn0%3D%7C3000&sdata=LVh8CSntvnHyFcjKHkKDxQZd89PtHpRIsgygYoMdglI%3D&reserved=0)). If published, this will include your full peer review and any attached files.

**>> We’re fine with this.**
